# Association between diet quality and risk of stunting among school-aged children in *Schistosoma mansoni* endemic area of western Kenya: a cross-sectional study

**DOI:** 10.1186/s41182-023-00566-0

**Published:** 2024-01-17

**Authors:** Madoka Kishino, Azumi Hida, Evans A. Chadeka, Manabu Inoue, Mayuko Osada-Oka, Sohkichi Matsumoto, Sammy M. Njenga, Shinjiro Hamano, Sachiyo Nagi

**Affiliations:** 1 Department of Food and Nutritional Science, Graduate School of Applied Bioscience, Tokyo University of Agriculture, Tokyo, Japan; 2 Department of Nutritional Science, Faculty of Applied Bioscience, Tokyo University of Agriculture, Tokyo, Japan; 3 Nagasaki University Institute of Tropical Medicine (NUITM) - Kenya Medical Research Institute (KEMRI), Nairobi, Kenya; 4 Kyokuto Pharmaceutical Industrial Co., Ltd, Tokyo, Japan; 5 Food Hygiene and Environmental Health Division of Applied Life Science, Graduate School of Life and Environmental Sciences, Kyoto Prefectural University, Kyoto, Japan; 6 Department of Bacteriology, Graduate School of Medical and Dental Sciences, Niigata University, Niigata, Japan; 7 Laboratory of Tuberculosis, Institute of Tropical Disease, Universitas Airlangga, Surabaya, East Java Indonesia; 8 Hokkaido University Institute for Vaccine Research and Development, Kita 20, Nishi 10, Kita-ku, Sapporo, Japan; 9 Eastern and Southern Africa Centre of International Parasite Control (ESACIPAC), Kenya Medical Research Institute (KEMRI), Nairobi, Kenya; 10 Department of Parasitology, Institute of Tropical Medicine (NEKKEN), Nagasaki University, Nagasaki, Japan; 11 The Joint Usage/Research Center on Tropical Disease, Institute of Tropical Medicine, Nagasaki University, Nagasaki, Japan; 12 Department of Hygiene and Public Health, Tokyo Women’s Medical University, Tokyo, Japan

**Keywords:** School-aged children, Malnutrition, Parasite infection, Diet quality, Dietary habit, Dietary guideline, Food Frequency Questionnaires, Kenya

## Abstract

**Background:**

Healthy eating habits are essential for improving nutritional status and strengthening immunity against infectious diseases. This study examined the relationship between diet quality and stunting in school-aged children in an infectious disease-endemic area of western Kenya.

**Methods:**

This cross-sectional study included 260 school-aged children (age 9–17 years) enrolled in primary schools in Mbita Sub-county, western Kenya. The nutritional status was assessed using anthropometric measurements. Dietary intake was measured using food frequency questionnaires and evaluated using the Food Pyramid (FP) score, which indicates adherence to the Kenyan food-based dietary guideline. Information on the children’s age, sex, maternal education, and household wealth index was collected using a household-based questionnaire. Infections with the predominant parasites, such as *Schistosoma (S.) mansoni,* were detected via microscopy. The trend associations of the FP score with food group intake were examined to characterize the dietary intake of this population. Logistic regression analysis was performed to investigate the relationship between stunting and FP score tertiles, adjusted for sociodemographic and economic indicators and parasitic infection status.

**Results:**

Among the studied schoolchildren, 15.0% exhibited stunting, while 76.2% were infected with *S. mansoni*. The mean FP score was 25.6 out of 50 points. A higher FP score was characterized by a high intake of roots and tubers, dairy products, pulses, and fruits and a low intake of cereals and animal-source foods. The analysis revealed a trend: a lower risk of stunting was evident in groups with elevated FP scores (*p* for trend = 0.065). However, these trend associations were observable among subjects with either negative or light *S. mansoni* infection (*p* for trend = 0.016).

**Conclusions:**

A higher quality diet, as evaluated by FP scores, was associated with a low risk of stunting among school-aged children. Notably, this association seemed to weaken in the presence of a high burden of *S. mansoni* infection. It highlights the importance of enhancing dietary quality through the promotion of diverse nutrient-dense foods alongside effective *S. mansoni* infection control for improved growth. This study contributes fundamental knowledge for understanding the diet–malnutrition relationship in areas endemic for *S. mansoni* infection.

**Supplementary Information:**

The online version contains supplementary material available at 10.1186/s41182-023-00566-0.

## Background

The arguments are clear regarding the need to invest in adolescent health [1, 2]. Moreover, investing in health has significant health benefits that can reduce future healthcare costs. However, a lack of data is a barrier to improving health, particularly in low- and middle-income countries [3]. Adolescents are at a critical stage of development. During adolescence, risky or protective behaviors such as eating and physical activity habits are initiated or reinforced. A study on primary school students in western Kenya suggested that height deficits relative to growth standards continued to increase with age [4]. Therefore, more studies are needed to elucidate the factors that affect growth faltering.

Schistosomiasis and soil-transmitted helminth (STH) infections are parasitic diseases affecting many school-aged children in sub-Saharan Africa [5–7]. In 2021, 251 million people worldwide were affected by schistosomiasis, of whom > 90% were living in sub-Saharan Africa and 54% were school-aged (5–14 years) children [5]. In 2021, 159 million school-aged children living in Africa will require preventive chemotherapy for STH [5]. Previous studies suggested high prevalence of schistosomiasis and undernutrition, such as wasting, stunting, or anemia, and their associations among school-aged children in sub-Saharan Africa [8–12]. Thus, the prevention of these parasitic diseases in school-aged children is an urgent challenge.

Multiple malnutrition burdens occur primarily due to poor diet [13]. Promoting healthy diets can contribute to better health and growth [14], strengthen immune systems, and prevent infectious diseases. However, studies on dietary intake among adolescents and school-aged children are scarce. Among these limited studies, high risks of micronutrient deficiencies, minimal intake of animal-source foods, and lack of dietary diversity were observed among adolescents and school-aged children [3, 15, 16].

The diet quality metric is a measure of dietary diversity, consumption of nutritionally important foods, and micronutrient adequacy and plays an essential role in understanding the risk factors for poor health outcomes rather than focusing on specific nutrients or foods [17–21]. The Kenya Food Pyramid (FP) is a dietary guideline for Kenyans that indicates what (food group-based) and how much should be consumed per day [22, 23]. Dietary evaluation by examining the adherence level to FP recommendations, called the FP score, can be used to help avoid nutrient insufficiency [24]. Examining the distribution of FP scores to characterize the population’s dietary intake and how socioeconomic backgrounds, such as household wealth index, affect the score is essential for planning strategies to improve malnutrition.

Despite the recognized negative impact of both poor diets and parasitemia on undernutrition [12, 25], there remains a gap in exploring the diet quality specifically among school-aged children residing in areas with high risk of parasite endemicity. Thus, this study seeks to fill this gap by investigating the correlation between diet quality indicators and nutritional status. By considering parasitic infections, it aims to explore discern tailored strategies for enhancing dietary practices within this population.

## Methods

### Study area

The study area was Mbita Sub-County, Homabay County, on the shores and islands of Lake Victoria in western Kenya. The study area is, under a health and demographic surveillance system (HDSS), Rusinga east and west on the island and Gembe east and west on the mainland. During the 2011 survey, the population of the Mbita HDSS was 55,929. Parasitic infections, especially schistosomiasis, are an urgent public health concern [26, 27]. Mbita is dominated by fishing communities near the lake. Temperatures range from 15 to 30 ℃ with a bimodal rainy season. The short rainy season occurs between October and December, whereas the long rainy season occurs from March to May. The average annual rainfall is 800–1200 mm in the western part of the study area on Rusinga Island, whereas Gembe receives 800–1900 mm.

### Study design and population

This was a secondary analysis of a cross-sectional study that explored malnutrition risk factors in the study population. The study was conducted between September and November 2011, at the end of the dry season, among fourth-grade primary school children. According to the education office in Mbita Sub-County, the primary school enrolment rate was 91.6% at the time of the survey. The schools included were full-grade primary schools with no history of mass chemotherapy 1 year before the study. Of the 64 public primary schools in the study area, 39 met the inclusion criteria. From these, eight schools were randomly selected. None of the selected schools was implementing school feeding programs. At the time of the survey, medications designed for treatment of schistosomiasis were not available at the study sites, save for their utilization in mass chemotherapy, and thus were not considered within the exclusion criteria. We enrolled 310 children, representing 98% of the children who met the inclusion criteria in the eight selected schools. We included children with complete anthropometric, parasitic infection and dietary data who were unlikely to have significant under- or over-reporting errors in daily energy intake.

### Anthropometric measurements

Anthropometric measurements were used to assess nutritional outcomes. Weight and height were measured at school using a Seca scale with a height rod (Seca 786; Seca GmbH & Co. KG, Hamburg, Germany). Body mass index (BMI) was calculated as the weight (kg) divided by the square of the height (m). The height-for-age Z score (HAZ) and the BMI-for-age Z score (BAZ) were calculated using AnthroPlus software from the World Health Organization [28]. Based on the WHO definitions, children with HAZ and BAZ less than − 2 were categorized as stunted and underweight, respectively.

### Dietary evaluation

Dietary intake was assessed using a food frequency questionnaire (FFQ) tailored to capture individual-level relative dietary patterns within this study population. The FFQ’s development involved preliminary 24-h diet recalls among ten local staff members to ensure its relevance. The FFQ included 41 food items asking about the frequency of consumption (nine options: never, 1–3 times a month, once a week, 2–4 times a week, 5–6 times a week, once a day, 2–3 times a day, 4–5 times a day, and > 6 times a day) and portion size. The enumerators conducted inquiries in Luo or Swahili, asking parents/guardians of the target children about the frequency and quantity of food items consumed in the past month. The amount was estimated as an approximate number of portions per meal, based on one portion using utensils such as bowls, tablespoons, teaspoons, plates, and cups. According to the answers in the FFQ, the daily intake of each item (g) was calculated as follows: number of portions × 1 portion amount × frequency. Daily energy and macronutrient intakes (energy ratios) were calculated using the Kenya food composition tables 2018 [29].

We evaluated diet quality based on the adherence level to the Kenyan food pyramid recommendations [22–24] called the FP score. In brief, the score was based on the recommended serving range for five food groups (staple foods, protein-rich foods, dairy products, vegetables, and fruits), as shown in Additional file 1: Table S1. Ten points were assigned if the number of servings consumed was within the recommended range. Fewer points were assigned for serving numbers outside the recommended range. The degree of adherence to the recommended serving range in the food guide pyramid was expressed as the sum of the scores of the five groups, each with a maximum of 10 points (maximum of 50 points). According to the Kenya food pyramid, we categorized the 41 food items in the FFQ into five food groups. The number of servings consumed for each food group was also calculated. For better interpretation, general starches were divided into two subgroups: (i) cereals and grains and (ii) potatoes, tubers, and starches. Protein-rich foods were also divided into two subgroups: (i) pulses and (ii) animal-source foods. The conversion of gram amounts to servings has been described previously [24].

### Parasitic tests for malaria and helminthic infections and hemoglobin assessment

Malarial and helminthic infections were examined in a previous report on this study population [26]. Stool samples were examined for the presence of *Schistosomiasis (S.) mansoni* and STH eggs using the Kato–Katz fecal thickness smear technique. *S. mansoni* infection was defined as at least one egg count on either day of examination. *S. mansoni* infection intensity was expressed as eggs per gram of feces (epg). Based on the WHO categorization, *S. mansoni* infections were categorized as light (1–99 epg), moderate (100–399 epg), or heavy (≥ 400 epg) [30]. The STHs examined were *Ascaris lumbricoides*, *Trichuris trichiura*, and hookworms. Thick blood smears were examined under a light microscope to detect malaria infections. Positive cases were defined as the detection of at least one malaria parasite in a microscopic field of 200 white blood cells on thick films or 2000 red blood cells on thin films [31].

For hemoglobin (Hb) assessment, we conducted compete blood count (CBC) using a Hemolyzer® 3NG machine (Analyticon Biotechnologies GmbH, Lichtenfels, Germany). After Hb determination, participants were categorized as being anemic based on the WHO’s threshold of < 11.5 g/dL Hb for children 5–11 years, < 12 g/dL Hb for children 12–14 years and nonpregnant females above 15 years, and < 13 g/dL Hb for males above 15 years [32].

### Questionnaire survey

Trained enumerators gathered information on children’s age, sex, mother/female guardian’s educational attainment, and household possessions from parents/guardians in home settings using a pretested questionnaire. Each child’s age was confirmed using official birth certificates or church baptism cards during household visits. In addition, an observation checklist was used to collect information on the house structure, latrines, and electricity availability in each household. Principal component analysis was conducted with variables including land ownership, electricity availability, latrines, and housing structure to create the household wealth index variable, which was categorized into tertiles of poorest, poorer, and least poor.

### Statistical analysis

The participants were classified into tertiles according to their FP scores (T1, T2, and T3). Food group intake was energy-adjusted using density methods, shown as servings per 1000 kcal. We examined the trend associations between FP score tertiles and energy-adjusted food group intake using the Jonckheere–Terpstra test to confirm the FP score in this specific study population. The trend associations of dietary intake by wealth index, which is thought to influence dietary intake, were also examined in Additional file 1.

Logistic regression analyses were used to examine the association between stunting and diet quality (FP score). The outcome (dependent variable) was stunting, and the FP score tertile was the explanatory (independent) variable. Age-adjusted and multivariate-adjusted odds ratio (OR) and 95% confidence interval (CI) of stunting were determined for each FP-score tertile relative to the first tertile (T1). We adjusted for the child’s age and sex, total energy intake, household wealth index, mother/female guardian educational attainment, *S. mansoni* infection status, coinfection of *S. mansoni* infection and malaria (*Plasmodium falciparum*), and anemia status, as these variables might affect both nutritional status and dietary intake [11, 25, 33]. We did not include STH infection status as a covariate because they were rare in this population. To delve deeper into the interlinkage between diet, parasitic infection, and risk of stunting, we conducted stratified analyses-based *S. mansoni* infection intensity (negative/light and moderate/heavy) [12]. In addition, trend associations were examined by treating the median FP score for each tertile as a continuous variable. Sex-stratified analysis was also performed as in Additional file 1. All statistical analyses were conducted using IBM SPSS Statistics for Windows, version 28.0 (IBM Corp., Armonk, NY, USA). The significance level was set at 5% for two-tailed tests.

## Results

Of the 310 studied children, we excluded children with no dietary data (*n* = 35) and those with a daily energy intake of < 500 kcal (*n* = 14) or > 4500 kcal (*n* = 1). This study included 260 fourth-grade primary school children with a mean age of 11.8 years (range 9–17 years) (Table 1). We note that some pupils exhibited delayed enrollment or progression, contributing to the wider age range observed. The FP scores ranged widely from 11 to 43 points out of a total possible score of 50 points. The ranges of FP scores were 11.5–22.8, 22.8–28.4, and 28.4–43.2 for T1–T3, respectively. At the time of the survey, 39 students (15.0%) had stunted growth, and 19 (7.3%) were underweight (Table 1). The prevalence of anemia was 40.2%. Most of the participants (76.2%) were infected with *S. mansoni*. Of these, 36.9%, 23.5%, and 15.8% exhibited light, moderate, and high infection intensities, respectively. The prevalence rates of malaria and STH infections were 11.5% and 13.5%, respectively.

**Table 1:** Characteristics of participants according to tertiles of FP score

	Overall (*N* = 260)	FP score tertiles		
		T1 (*n* = 86)	T2 (*n* = 87)	T3 (*n* = 87)
FP score^a^	25.6 ± 6.3	18.5 ± 3.1	25.9 ± 1.6	32.3 ± 3.2
Sex				
Boys	116 (44.6)	32 (37.2)	46 (52.9)	38 (43.7)
Girls	144 (55.4)	54 (62.8)	41 (47.1)	49 (56.3)
Age (years)	11.8 ± 1.6	11.8 ± 1.5	11.6 ± 1.6	12.1 ± 1.7
9–11	117 (45.0)	39 (45.3)	41 (47.1)	37 (42.5)
12–14	129 (49.6)	45 (52.3)	42 (48.3)	42 (48.3)
15–17	14 (5.4)	2 (2.3)	4 (4.6)	8 (9.2)
Household wealth status				
Poorest	101 (38.8)	40 (46.5)	32 (36.8)	29 (33.3)
Poorer	79 (30.4)	22 (25.6)	29 (33.3)	28 (32.2)
Least poor	89 (30.8)	24 (27.9)	26 (29.9)	30 (34.5)
Mother’s/female guardian’s education				
Higher than primary education (> 8 years)	100 (38.5)	30 (34.9)	36 (41.4)	34 (39.1)
< 8 years	160 (61.5)	56 (65.1)	51 (58.6)	53 (60.9)
HAZ	− 0.63 ± 1.23	− 0.7 ± 1.26	− 0.58 ± 1.25	− 0.62 ± 1.19
Stunting (HAZ < − 2)	39 (15.0)	16 (18.6)	12 (13.8)	11 (12.6)
BAZ	− 0.77 ± 0.84	− 0.72 ± 0.89	− 0.72 ± 0.77	− 0.85 ± 0.87
Underweight (BAZ < − 2)	19 (7.3)	8 (9.3)	5 (5.7)	6 (6.9)
Anemia^b^	101 (40.2)	36 (42.4)	36 (42.4)	29 (35.8)
*S. mansoni* infection				
Light	98 (36.9)	28 (32.6)	37 (42.5)	31 (35.6)
Moderate	61 (23.5)	28 (32.6)	19 (21.8)	14 (16.1)
Heavy	41 (15.8)	11 (12.8)	14 (16.1)	16 (18.4)
Malaria infection	30 (11.5)	11 (12.8)	8 (9.2)	11 (12.6)
*S. mansoni* and malaria coinfection	24 (9.2)	10 (11.6)	5 (5.7)	9 (10.3)
Soil-transmitted helminth infection	35 (13.5)	11 (12.8)	13 (14.9)	11 (12.6)

*T* tertile, *FP* Kenya Food Pyramid, *HAZ* height-for-age Z score, *BAZ* BMI (body mass index)-for-age Z score, *S. mansoni* *Schistosoma mansoni*

Data are shown as the mean ± SD or number (percentage)

^a^FP score indicates adherence to the Kenyan Food Pyramid

^b^Nine cases were missing

As shown in Table 2, a significant trend was observed for the FP score and energy intake and protein-energy ratio, in which the higher the FP score from T1 to T3, the higher the energy intake and the lower the protein-energy ratio. In addition, a higher FP score indicated a higher intake (servings per 1000 kcal) of roots, tubers, and starches, dairy products, and fruits and a lower intake of cereals and grains, protein-rich foods, and animal-source foods (*p* for trend < 0.05). The sex-stratified analysis showed that food group intake, characterized by the FP score, varied by sex (Additional file 1: Tables S2 and S3). Higher FP scores indicated a higher intake of roots, tubers, and starches, dairy products, fruits, and sugar among boys and a higher intake of roots, tubers, and starches, dairy products, pulses, vegetables, and fruits among girls. Higher wealth indices were associated with higher intakes of roots, tubers, and starches, and fruits and lower intakes of cereals and grains (Additional file 1: Table S4).

**Table 2:** Trend associations of FP score tertiles with energy and food intakes among school-aged children, Mbita Sub-County, western Kenya

	Overall (*N* = 260)	FP score^a^ tertiles			*p* for trend*
		T1 (*n* = 86)	T2 (*n* = 87)	T3 (*n* = 87)	
Total energy (kcal/day)	1298 ± 463	982 ± 372	1331 ± 401	1578 ± 408	< 0.001
Protein-energy ratio (% of energy)	19.8 ± 4.8	20.8 ± 6.2	19.9 ± 4.1	18.7 ± 3.7	0.018
Fat-energy ratio (% of energy)	27.8 ± 8.1	28.0 ± 9.1	27.3 ± 8.3	28.0 ± 6.7	0.560
Carbohydrate-energy ratio (% of energy)	52.4 ± 9.4	51.2 ± 11.6	52.7 ± 8.9	53.3 ± 7.4	0.381
Energy-adjusted food group intake (SV/1000 kcal)					
General starches	4.7 ± 1.4	4.7 ± 1.8	4.8 ± 1.3	4.5 ± 1.1	0.350
Cereals and grains	4.3 ± 1.5	4.6 ± 1.8	4.5 ± 1.4	4.0 ± 1.2	0.023
Roots, tubers, and starches	0.3 ± 0.5	0.1 ± 0.3	0.3 ± 0.5	0.5 ± 0.6	< 0.001
Dairy products	0.2 ± 0.4	0.1 ± 0.3	0.2 ± 0.4	0.4 ± 0.5	< 0.001
Protein-rich foods	4.1 ± 1.9	4.5 ± 2.5	4.0 ± 1.5	3.7 ± 1.4	0.040
Pulses	0.3 ± 0.4	0.31 ± 0.47	0.29 ± 0.35	0.34 ± 0.36	0.006
Animal-source foods	3.7 ± 1.9	4.2 ± 2.5	3.7 ± 1.5	3.3 ± 1.3	0.028
Vegetables	1.1 ± 0.8	1.0 ± 0.6	1.2 ± 0.9	1.2 ± 0.9	0.080
Fruits	0.8 ± 0.8	0.6 ± 0.8	0.7 ± 0.7	1.1 ± 0.7	< 0.001
Fat	10.6 ± 16.7	13.3 ± 19.5	8.2 ± 11.8	10.3 ± 17.5	0.319
Sugar	2.8 ± 3.8	2.9 ± 4.3	2.2 ± 3.3	3.3 ± 3.8	0.067

Data are presented as mean ± SD

*T* tertile, *FP* Kenya Food Pyramid

^a^FP score indicates adherence to the Kenyan Food Pyramid

^*^p values were based on the Jonckheere–Terpstra test

In the multivariable-adjusted model, encompassing parasitic infections, the ORs for stunting across FP score tertiles—using tertile 1 as a reference—were 0.57 (95% CI 0.23–1.44) for tertile 2 and 0.36 (95% CI 0.12–1.09) for tertile 3 (*p* for trend = 0.065) (Table 3). Upon stratification by *S.mansoni* infection intensity, the ORs for stunting were 0.19 (95% CI 0.05–0.76) for tertile 2 and 0.17 (95% CI 0.04–0.77) for tertile 3 (*p* for trend = 0.016) in subjects with negative/light infection, and 1.63 (95% CI 0.31–8.61) for tertile 2 and 2.55 (95% CI 0.31–20.95) for tertile 3 (*p* for trend = 0.355) in subjects with moderate/heavy infection. Notably, in sex-stratified analyses (Additional file 1: Table S5), these associations remained significant solely among girls.

**Table 3:** Odds ratios of stunting according to FP score tertile^a^ among school-aged children, Mbita Sub-County, western Kenya

	T1		T2		T3		*p* for trend^b^
Total (*n* = 260)							
Stunting, no. (%)	16	(18.6)	12	(13.8)	11	(12.6)	
Age-adjusted OR (95%CI)	1	[Reference]	0.72	(0.32–1.64)	0.59	(0.25–1.36)	0.206
Multivariable adjusted OR (95%CI)^c,d^	1	[Reference]	0.57	(0.23–1.44)	0.36	(0.12–1.09)	0.065
Subjects with negative or light *Schistosoma mansoni* infection (*n* = 158)							
Stunting, no. (%)	11	(23.4)	5	(9.3)	7	(12.3)	
Age-adjusted OR (95%CI)	1	[Reference]	0.33	(0.10–1.05)	0.44	(0.16–1.27)	0.105
Multivariable adjusted OR (95%CI)^c,d^	1	[Reference]	0.19	(0.05–0.76)	0.17	(0.04–0.77)	0.016
Subjects with moderate or heavy *Schistosoma mansoni* infection (*n* = 102)							
Stunting, no. (%)	5	(12.8)	7	(21.2)	4	(13.3)	
Age-adjusted OR (95%CI)	1	[Reference]	1.91	(0.54–6.8)	0.90	(0.21–3.88)	0.982
Multivariable adjusted OR (95%CI)^c,d^	1	[Reference]	1.63	(0.31–8.61)	2.55	(0.31–20.95)	0.355

*T* tertile, *FP* food pyramid, *OR* odds ratio, *CI* confidence interval

^a^FP score indicates adherence to the Kenyan Food Pyramid

^b^Median FP score for each tertile was treated as an independent variable (continuous)

^c^Multivariable analysis was adjusted for age (continuous), sex, energy intake (continuous), wealth index (the poorest, poorer, the least poor), mother's/female guardian’s education [less than primary or higher than primary (8 years)], *S. mansoni* infection intensity (negative, light, moderate, and heavy), coinfection of *S. mansoni* and malaria (yes or no), and anemia prevalence (yes or no)

^d^Nine participants with missing information on anemia were excluded from the analyses

## Discussion

This cross-sectional study examined the association between diet quality, as measured by adherence to the Kenyan Food Pyramid’s recommendations (FP score), and stunting in school-aged children living in areas at high risk of *S. mansoni* infection. Students with higher FP scores consumed more energy-adjusted roots, tubers and starches; dairy products; pulses; and fruits compared to the tertile with the lowest FP score. The study found that the higher FP score, the lower ORs for stunting. Thus, this study suggests a higher quality diet, as evaluated by FP score, contribute children’s improved growth.

In this study population, the prevalence of *S.mansoni* infection marked at 76.2% (95%CI 70.5–81.2%), with 39.2% (95%CI 33.3–45.5%) showing moderate to heavy infection during the 2011 survey. We conducted a rigorous detection process by examining fecal specimens over 3 consecutive days to ensure optimal detection of *S. mansoni* infection. A separate study highlighted a significant nationwide reduction in the prevalence of STHs and schistosome infections following Kenya’s implementation of a national school-based deworming program in 2012 [34]. Despite this, recent reports indicate persistently high schistosome infection rates in our study sites, surpassing 50% on Rusinga island (unpublished information). The World Health Organization (WHO) has set a target to eliminate heavy intensity schistosome infections by 2030 [35], signaling a need for intensified efforts to alleviate the burden of parasite infections in the near future.

The prevalence of stunting among school-aged children in this study was 15.0%, which was similar to or lower, considering the high *S. mansoni* infection rate. For example, in a 2017 Tanzanian study, *S. mansoni* infection rate was 90.6% and stunting rate was 29.0% [8]. In a 2011 survey in Ethiopia, the *S. mansoni* infection rate was 53.2% and stunting rate was 11.2% [10].

Our analyses revealed that the group with the high FP score, characterized by a high intake of roots, tubers, and starches, dairy products, and fruits, had a lower risk of stunting. This emphasizes the pivotal role of enhancing diet quality in combating stunting. A previous study showed that higher FP scores were associated with lower the risk of nutrient insufficiency among adults [24]. These findings are partly consistent with previous reports of the significant association of healthy and diverse food group intake with a lower risk of stunting in school-aged children in China [36]. Studies examining dietary intake among school-aged children also suggested that limited diverse diets and insufficient micronutrient intake were major challenges [16, 37]. In addition, the results of the present study showed associations between wealth index and the intake of roots, tubers and starches, and fruits. Wealthier households may have better access to these foods, that are essential sources of carbohydrates and micronutrients in the study area. An approach to endure sufficient amount of diverse healthy foods is recommended. Further investigations to enhance the quality of diets among school-aged children are needed.

The lower risk of stunting due to higher diet quality was only observed in those with a low burden of *S. mansoni* infection. Our results also suggested a potential attenuation of the protective effect of high-quality diets on nutritional status in the presence of a burdensome *S. mansoni* infection. The adverse gastrointestinal effects of intestinal schistosomiasis such as abdominal pain, diarrhea, blood in the stool [35] can impede nutrient absorption, contributing to growth faltering. Previous studies have also evidenced a higher risk of stunting among school-aged children with heavier *S. mansoni* infection [8, 12]. Urgent interventions targeting the elimination of heavy-intensity schistosomiasis, as prioritized by the WHO [35], become imperative in this context.

In addition, sex-stratified analysis also revealed the difference associations, indicating that the underlying cause of malnutrition may also vary by sex. Furthermore, the consumption of food groups characterized by the FP score also varied by sex, suggesting the presence of gender differences in dietary intake patterns or reporting errors as suggested in previous studies too [37, 38].

To our knowledge, this is the first study to evaluate diet quality among school-aged children and examine its association with nutritional status after adjusting for household socioeconomic and *S. mansoni* infection status. This study focused on school-aged children, a second window of opportunity for growth, psychosocial development, and establishing lifelong dietary and lifestyle habits. The indicators used in this study, based on the Kenyan food-based dietary guidelines (FP score), were suggested to be useful for assessing diet quality in school-aged children.

However, our study has some limitations. First, the FFQ has not yet been validated. Therefore, the FFQ must be validated in future studies. Despite these limitations, our focus in the FFQ allowed us to capture food variety and relative intake, revealing associations between certain dietary patterns and a reduced risk of stunting. The findings indicated that children reporting higher consumption of roots and tubers, dairy products, pulses and fruits showed a lower risk of stunting. However, the extent of potential reporting errors remains uncertain. In addition, reliance on parental/guardian recall for reporting children’s dietary intake may introduce recall and reporting biases. Finally, an unknown causal relationship was present due to the cross-sectional study design. Although the FFQ is intended to capture habitual intake, the period of dietary evaluation, a month, might not be sufficient to relate to stunting resulting from chronic or recurrent undernutrition.

## Conclusion

This cross-sectional study of school-aged children in an *S. mansoni* infection-endemic area found that a better diet, as per the FP score aligned with the Kenya Food Pyramid, reduced the likelihood of stunting. However, the presence of a high burden of *S. mansoni* infections could weaken this dietary effect. Given the observed positive association between the FP score, intake of diverse nutritious foods, and wealth index, an approach to improve access to these foods would be recommended. These findings provide a basis for understanding diet quality and associated factors for better growth among school-aged children in *S. mansoni* endemic areas.

### Supplementary Information

**Additional file 1: Table S1.** Definition of 1SV and the FP score criteria for each food group. **Table S2.** Trend association of FP score tertiles with energy and food intakes of boys, school children in Mbita Sub-County, Western Kenya. **Table S3.** Trend association of FP score tertiles with energy and food intakes of girls, school children in Mbita Sub-County, Western Kenya. **Table S4.** Dietary intake according to household wealth status, school children in Mbita Sub-County, Western Kenya. **Table S5.** Odds ratios of stunting according to tertiles of the FP score by sex, school children in Mbita Sub-County, Western Kenya.

## Data Availability

The datasets used and/or analyzed in the current study are available from the corresponding author upon reasonable request.

## Abbreviations

BAZ: BMI-for-age Z score
BMI: Body mass index
CI: Confidence interval
FFQ: Food Frequency Questionnaire
FP: Food pyramid
HAZ: Height-for-age z score
OR: Odds ratio
*S. mansoni*: *Schistosoma mansoni*
STH: Soil-transmitted helminth
T: Tertile
WHO: World Health Organization
