# Supplementary material for: Association between diet quality and risk of stunting among school-aged children in Schistosoma mansoni endemic area of western Kenya: a cross-sectional study
Source: Trop Med Health. 2024 Jan 17;52:12. doi: 10.1186/s41182-023-00566-0 (PMC10792916; doi:10.1186/s41182-023-00566-0)
Supplement: Supplementary file 1 — Additional file 1: Table S1. Definition of 1SV and the FP score criteria for each food group. Table S2. Trend association of FP score tertiles with energy and food intakes of boys, school children in Mbita Sub-County, Western Kenya. Table S3. Trend association of FP score tertiles with energy and food intakes of girls, school children in Mbita Sub-County, Western Kenya. Table S4. Dietary intake according to household wealth status, school children in Mbita Sub-County, Western Kenya. Table S5. Odds ratios of stunting according to tertiles of the FP score by sex, school children in Mbita Sub-County, Western Kenya. [file 41182_2023_566_MOESM1_ESM.docx]

Table S1

Definition of 1SV and the FP score criteria for each food group

| Food group^1)^ | Definition of 1SV^2)^ | Recommended SVs^3)^ (SVs / day) | FP score |
| --- | --- | --- | --- |
| General starches | 20 g Carbohydrate | 6 - 11 | **0 - 10** |
| Cereals and grains |  |  |  |
| Roots and tubers |  |  |  |
| Milk products | 300 mg Ca | 2 – 3 | **0 - 10** |
| Protein-rich foods | 6 g Protein | 5 - 7 | **0 – 10** |
| Plant-based foods |  |  |  |
| Animal-based foods |  |  |  |
| Vegetables | 80g weight | ≧3 | **0 – 10** |
| Green leafy vegetables |  |  |  |
| Other vegetables |  |  |  |
| Fruits | 100g weight | 2 - 3 | **0 – 10** |
| **Total** |  |  | **0 - 50** |
| FP, food pyramid; SV, serving, 1) The number of SVs was also calculated in sub-categories to examine the quality of the diet. 2) Defined in the present study. 3) Defined by Kenya National Clinical Nutrition and Dietetics Reference Manual, 2010 | | | |

Table S2

Trend association of FP score tertiles with energy and food intakes of boys, schoolchildren in Mbita Sub-County, Western Kenya

|  | Overall (n=116) | | | FP score tertiles | | | | | | | | | p for trend* |
| --- | --- | --- | --- | --- | --- | --- | --- | --- | --- | --- | --- | --- | --- |
|  |  |  |  | T1 (n=32) | | | T2 (n=46) | | | T3 (n=38) | | |  |
| Total energy (kcal/day) | 1375 | ± | 474 | 1036 | ± | 437 | 1396 | ± | 396 | 1636 | ± | 421 | <0.001 |
| Protein energy ratio (% of energy) | 19.9 | ± | 4.7 | 21.3 | ± | 6.2 | 19.9 | ± | 4.0 | 18.6 | ± | 3.6 | 0.052 |
| Fat energy ratio (% of energy) | 27.9 | ± | 7.9 | 28.7 | ± | 8.6 | 28.6 | ± | 7.2 | 26.3 | ± | 8.0 | 0.147 |
| Carbohydrate energy ratio (% of energy) | 52.3 | ± | 9.6 | 50.0 | ± | 11.4 | 51.5 | ± | 8.7 | 55.1 | ± | 8.6 | 0.047 |
| Energy-adjusted food group intake (SV/1000kcal) | |  |  |  |  |  |  |  |  |  |  |  |  |
| General starches | 4.6 | ± | 1.4 | 4.4 | ± | 1.7 | 4.6 | ± | 1.3 | 4.8 | ± | 1.2 | 0.200 |
| Cereals and grains | 4.3 | ± | 1.4 | 4.2 | ± | 1.8 | 4.3 | ± | 1.4 | 4.4 | ± | 1.2 | 0.551 |
| Roots, tubers and starches | 0.3 | ± | 0.5 | 0.2 | ± | 0.3 | 0.3 | ± | 0.4 | 0.5 | ± | 0.6 | <0.001 |
| Dairy products | 0.3 | ± | 0.5 | 0.2 | ± | 0.3 | 0.2 | ± | 0.5 | 0.4 | ± | 0.5 | 0.014 |
| Protein-rich foods | 4.0 | ± | 1.8 | 4.5 | ± | 2.5 | 3.9 | ± | 1.5 | 3.5 | ± | 1.3 | 0.054 |
| Pulses | 0.3 | ± | 0.3 | 0.3 | ± | 0.5 | 0.3 | ± | 0.3 | 0.3 | ± | 0.2 | 0.317 |
| Animal-source foods | 3.7 | ± | 1.8 | 4.2 | ± | 2.5 | 3.7 | ± | 1.5 | 3.2 | ± | 1.3 | 0.072 |
| Vegetables | 1.1 | ± | 0.8 | 1.0 | ± | 0.7 | 1.1 | ± | 0.7 | 1.0 | ± | 0.9 | 0.632 |
| Fruits | 0.7 | ± | 0.7 | 0.5 | ± | 0.7 | 0.7 | ± | 0.7 | 1.1 | ± | 0.7 | <0.001 |
| Fat | 9.6 | ± | 16.1 | 10.5 | ± | 16.3 | 6.9 | ± | 7.1 | 12.2 | ± | 22.5 | 0.445 |
| Sugar | 2.8 | ± | 3.9 | 2.4 | ± | 3.8 | 2.4 | ± | 3.7 | 3.7 | ± | 4.1 | 0.049 |
| Data are shown as mean±SD, *p-values were based on Jonckheere-Terpstra test. | | | | | | | |  |  |  |  |  |  |

Table S3

Trend association of FP score tertiles with energy and food intakes of girls, schoolchildren in Mbita Sub-County, Western Kenya

|  | Overall (n=144) | | | FP score tertiles | | | | | | | | | p for trend* |
| --- | --- | --- | --- | --- | --- | --- | --- | --- | --- | --- | --- | --- | --- |
|  |  |  |  | T1 (n=54) | | | T2 (n=41) | | | T3 (n=49) | | |  |
| Total energy (kcal/day) | 1236 | ± | 445 | 949 | ± | 328 | 1258 | ± | 398 | 1533 | ± | 395 | <0.001 |
| Protein energy ratio (% of energy) | 19.8 | ± | 5.0 | 20.6 | ± | 6.3 | 20.1 | ± | 4.3 | 18.7 | ± | 3.8 | 0.152 |
| Fat energy ratio (% of energy) | 27.7 | ± | 8.3 | 27.5 | ± | 9.5 | 25.9 | ± | 9.2 | 29.3 | ± | 5.3 | 0.084 |
| Carbohydrate energy ratio (% of energy) | 52.6 | ± | 9.3 | 51.9 | ± | 11.7 | 54.1 | ± | 9.0 | 52.0 | ± | 6.0 | 0.583 |
| Energy-adjusted food group intake (SV/1000 kcal) | |  |  |  |  |  |  |  |  |  |  |  |  |
| General starches | 4.7 | ± | 1.5 | 4.8 | ± | 1.9 | 5.0 | ± | 1.3 | 4.3 | ± | 0.9 | 0.039 |
| Cereals and grains | 4.4 | ± | 1.6 | 4.7 | ± | 1.9 | 4.7 | ± | 1.4 | 3.7 | ± | 1.0 | 0.001 |
| Roots, tubers and starches | 0.3 | ± | 0.5 | 0.1 | ± | 0.2 | 0.3 | ± | 0.5 | 0.6 | ± | 0.7 | <0.001 |
| Dairy products | 0.2 | ± | 0.3 | 0.1 | ± | 0.3 | 0.1 | ± | 0.2 | 0.3 | ± | 0.4 | <0.001 |
| Protein-rich foods | 4.2 | ± | 2.0 | 4.5 | ± | 2.6 | 4.1 | ± | 1.6 | 3.8 | ± | 1.5 | 0.249 |
| Pulses | 0.3 | ± | 0.5 | 0.31 | ± | 0.48 | 0.32 | ± | 0.44 | 0.41 | ± | 0.42 | 0.005 |
| Animal-source foods | 3.8 | ± | 1.9 | 4.2 | ± | 2.5 | 3.8 | ± | 1.6 | 3.4 | ± | 1.4 | 0.157 |
| Vegetables | 1.2 | ± | 0.9 | 0.9 | ± | 0.6 | 1.3 | ± | 1.1 | 1.4 | ± | 0.9 | 0.006 |
| Fruits | 0.8 | ± | 0.8 | 0.6 | ± | 0.9 | 0.7 | ± | 0.8 | 1.1 | ± | 0.7 | <0.001 |
| Fat | 11.3 | ± | 17.1 | 15.0 | ± | 21.2 | 9.6 | ± | 15.4 | 8.8 | ± | 12.4 | 0.076 |
| Sugar | 2.8 | ± | 3.8 | 3.2 | ± | 4.6 | 1.9 | ± | 2.7 | 3.0 | ± | 3.6 | 0.498 |
| Data are shown as mean±SD, *p-values were based on Jonckheere-Terpstra test. | | | | | | | |  |  |  |  |  |  |

Table S4

Dietary intake according to household wealth status, schoolchildren in Mbita Sub-County, Western Kenya

|  | Wealth status | | | | | | | | | |  |
| --- | --- | --- | --- | --- | --- | --- | --- | --- | --- | --- | --- |
|  | Poorest (n=101) | | Poorer (n=79) | | | | Least poor (n=80) | | | |  |
|  | mean | SD | mean | | SD | | mean | | SD | | P for trend* |
| FP score | 25.0 | 6.6 | 25.6 | | 6.0 | | 26.3 | | 6.1 | | 0.158 |
| Total energy (kcal/day) | 1261 | 464 | 1329 | | 482 | | 1315 | | 444 | | 0.223 |
| Protein energy ratio (%E) | 19.9 | 4.6 | 19.7 | | 4.4 | | 19.8 | | 5.5 | | 0.506 |
| Fat energy ratio (%E) | 28.2 | 9.0 | 26.5 | | 7.4 | | 28.4 | | 7.4 | | 0.813 |
| Carbohydrate energy ratio (%E) | 51.8 | 9.9 | 53.8 | | 8.7 | | 51.8 | | 9.6 | | 0.646 |
| Energy-adjusted food group intake (SV/1000kcal) | | | | | | | | | | | |
| General starches | 4.7 | 1.5 | 4.9 | | 1.4 | | 4.4 | | 1.4 | | 0.420 |
| Cereals and grains | 4.5 | 1.6 | 4.6 | | 1.5 | | 4.0 | | 1.4 | | 0.038 |
| Roots, tubers and starches | 0.2 | 0.4 | 0.4 | | 0.4 | | 0.5 | | 0.6 | | 0.001 |
| Dairy products | 0.3 | 0.5 | 0.1 | | 0.2 | | 0.2 | | 0.4 | | 0.572 |
| Protein-rich foods | 4.1 | 1.9 | 4.0 | | 1.8 | | 4.1 | | 2.0 | | 0.945 |
| Pulses | 0.3 | 0.5 | 0.4 | | 0.4 | | 0.3 | | 0.3 | | 0.408 |
| Animal-source foods | 3.8 | 1.9 | 3.7 | | 1.7 | | 3.8 | | 2.0 | | 0.934 |
| Vegetables | 1.1 | 0.8 | 1.2 | | 1.0 | | 1.2 | | 0.8 | | 0.165 |
| Fruits | 0.7 | 0.9 | 0.7 | | 0.7 | | 0.9 | | 0.8 | | 0.038 |
| Fat | 12.8 | 18.5 | 7.2 | | 9.2 | | 11.2 | | 19.4 | | 0.838 |
| Sugar | 2.7 | 3.8 | 2.3 | | 3.1 | | 3.4 | | 4.5 | | 0.445 |
| *P-values were based on Jonckheere-Terpstra test | | | |  | |  | |  | |  |  |

Table S5

Odds ratios of stunting according to tertiles of the FP score^1)^ by sex, schoolchildren in Mbita Sub-County, Western Kenya

|  | Tertile 1 | | Tertile 2 | | Tertile 3 | |
| --- | --- | --- | --- | --- | --- | --- |
| Boys (n=111) |  |  |  |  |  |  |
| FP score^1)^, mean (SD) | 18.1 | (3.4) | 25.8 | (1.6) | 31.6 | (3.4) |
| Stunting, no. (%) | 10 | (31.3) | 6 | (13.3) | 5 | (14.7) |
| Age-adjusted OR (95%CI) | 1 | [Reference] | 0.33 | (0.11-1.04) | 0.33 | (0.09-1.15) |
| Multivariable adjusted OR (95%CI)^2)^ | 1 | [Reference] | 0.31 | (0.09-1.06) | 0.38 | (0.10-1.51) |
| Girls (n=140) |  |  |  |  |  |  |
| FP score^1)^, mean (SD) | 18.9 | (2.8) | 25.8 | (1.6) | 32.8 | (3.2) |
| Stunting, no. (%) | 6 | (11.3) | 6 | (15.0) | 3 | (6.4) |
| Age-adjusted OR (95%CI) | 1 | [Reference] | 1.47 | (0.43-5.02) | 0.52 | (0.12-2.23) |
| Multivariable adjusted OR (95%CI)^2)^ | 1 | [Reference] | 1.09 | (0.29-4.10) | 0.40 | (0.09-1.89) |

T; Tertile, FP; Food pyramid, OR; Odds ratio, CI; Confidence interval, ^1)^ FP score indicates adherence to the Kenyan Food Pyramid. ^2)^ Multivariable analysis was adjusted for age (continuous), wealth index (poorest, poorer, and least poor), mother's/female guardian’s education (less than primary or higher than primary (8 years)), *S.mansoni* infection intensity (negative, light, moderate and heavy), coinfection of *S.mansoni* and malaria (yes or no) and anemia prevalence (yes or no). Nine participants with missing information on anemia were excluded from the analyses.
